# Spirulina supplementation to alleviate negative effects of lead in layer chicken

**DOI:** 10.5455/javar.2023.j735

**Published:** 2023-12-31

**Authors:** Md. Mizanur Rahman, Md. Shahidul Islam, Rakibul Hasan, Pritam Saha, Mohammad Shah Alam

**Affiliations:** 1 Department of Physiology and Pharmacology, Bangabandhu Sheikh Mujibur Rahman Agricultural University, Gazipur, Bangladesh; 2 Department of Pharmacology, Bangladesh Agricultural University, Mymensingh, Bangladesh; 3 Department of Anatomy and Histology, Bangabandhu Sheikh Mujibur Rahman Agricultural University, Gazipur, Bangladesh

**Keywords:** Spirulina, hematology, lead toxicity, laying hen

## Abstract

**Objectives:**

Lead (Pb), a toxic heavy metal, is a serious concern for poultry that negatively affects their productivity and health. To combat those issues efficiently, it is necessary to include feed supplements that have rich antioxidant properties for satisfactory health and productivity. *Spirulina platensis* (Sp), a microalgae, is a compound that provides several health benefits for humans and animals. This study explores that supplementation of Sp in diet as well as in water reduces the burden of Pb in different tissues, improves hematology, and improves the productive performance of advanced-age laying hens.

**Materials and methods:**

Forty birds were separated into four groups: the control (C), Spirulina (Sp), Pb, and (Pb + Sp) groups. The Pb group was given Pb acetate at a dose of 2 gm/l in water *ad libitum* for 4 weeks. Sp group was fed Sp at a dose of 4 gm/kg feed. The Pb + Sp group was given Pb and Sp as in the previous groups.

**Results:**

Productive performance and hematology such as hemoglobin (Hb), packed cell volume, red blood cell, mean corpuscular volume, mean corpuscular Hb (MCH) concentration, and MCH were significantly (*p <* 0.05) decreased in Pb-treated groups compared to controls. The distribution of Pb concentration was highest in the bones and lowest in the gizzard. However, Sp treatment significantly (*p <* 0.05) increased the productive performance and the hematological parameters. Moreover, Pb concentration in different organs significantly decreased in the group treated with Sp.

**Conclusion:**

This study indicates that Sp can possibly be used as a natural and powerful dietary additive to mitigate heavy metal intoxication in chickens, thereby being efficient and effective for production.

## Introduction

Lead (Pb), a toxic heavy metal, is the most established environmental pollutant and is considered a worldwide occupational, industrial, and environmental contaminant that influences a wide range of physio-biochemical and behavioral dysfunctions. Exposure to Pb is expected through soil, water, air, and industrial pollution and can easily enter the body through different routes [1]. After being absorbed in the blood, a part of Pb binds to the proteins of erythrocytes and inhibits hematopoiesis as well as the heme biosynthesis process, and at the same time, another part is distributed to accumulate in various tissues such as the bone, kidney, liver, and brain. Accumulated Pb cannot be metabolized in the body, and it is first and foremost excreted through glomerular filtration in the kidney and also through bile, pancreatic juice, and feces. A minor part is excreted through sweat, seminal fluid, nails, and also milk [2]. Excess bioaccumulation in visceral organs causes systemic function disturbances, including neurotoxicity, nephrotoxicity, and hepatotoxicity. Even at low concentrations, a number of physiological and biochemical actions are associated at the molecular and cellular level that can result in morphological deformities that cannot be reversed even after the levels of Pb have diminished in the body [3]. Consequently, various organs and systems, such as the hematopoietic, digestive, nervous, urinary, cardiovascular, and reproductive systems, can be affected by Pb intoxication. Moreover, Pb can affect the growth and development of broilers [4]. Furthermore, it can also affect the reproductive performance of chickens by affecting the endocrine system through increased oxidative stress [5].

Microalgae, *Spirulina platensis* (sp), is rich in antioxidants such as β-carotene, riboflavin, α-tocopherol, and α-lipoic acid and superoxide dismutase that scavenge free radicals, including superoxide, hydrogen peroxide, and hydroxyl radicals, thereby protecting body organs from oxidative stress caused by Pb exposure in rats [6]. Furthermore, Sp is a sustainable source of energy and is used commercially as an herbal medicine worldwide due to its low cost and minimal side effects [7]. Recently, dietary supplementation of microalgae, including Sp, has been shown to play a beneficial role in preventing or managing hypercholesterolemia, hyperglyceridemia, obesity, inflammation, cancer, and cardiovascular disease [8,9]. Moreover, it has been reported that Sp could enhance the growth, productivity, and reproductive accomplishment of animals and poultry through improved feed intake, feed conversion ratio, and utilization of nutrients, and subsequently increase body weight gain, carcass yield, egg weight, egg mass, egg component, egg quality, and hatchability [10]. Since Sp has multifaceted health benefits in animals, this study was designed to inquire into the potential ameliorative role of Sp in Pb-intoxicated advanced-age layer chickens. We explored the effect of Sp on hematology, Pb clearance, and productive performance in Pb-intoxicated advanced-age layer chickens.

## Materials and Methods

### Ethical statement

The research was carried out at the Department of Physiology and Pharmacology, BSMRAU. The experimental design was approved by the Institutional Animal Research Ethics Committee (AREC), BSMRAU, Gazipur-1706, Bangladesh. Ref. No. FVMAS/AREC/2023/17.

### Experimental birds, husbandry, diet, and treatment

Forty white leghorn layer chickens aged 60–65 weeks and body weight 1.4–1.5 kg/bird collected from Diamond Bangladesh Ltd., Dhaka, were used for this investigation. The layer chickens were housed in a laying case individually at a temperature of 25°C ± 1°C, in the humidity-controlled room, and a 13 h light and 11 h dark period was maintained for a 24 h lighting cycle. Commercial layer food (Nourish layer feed, Dhaka, Bangladesh) and tap water were provided *ad libitum* during a 1-week adaptation period. The chickens were then classified into four groups (*n =* 10) and designated as control (C), Spirulina (Sp), Pb, and Pb + Sp. The control group was given normal feed and water during the experiment period. The Sp group was fed normal feed supplemented with Sp at a dose of 4 gm/kg feed for 4 weeks. The Pb group was fed 2 gm/l of Pb acetate in water and the normal diet for 4 weeks. The Pb + Sp group was given Pb and Sp as used in the previous groups. *Spirulina platensis* and Pb were purchased from SK + F pharmaceutical company, Bangladesh Ltd., and Sigma Chemical Co., respectively.

### Sample collection

At the end of the treatment period, tissues from different organs such as bone, gizzard, liver, and heart of five birds from each group were collected and preserved for measurement of Pb concentration, and blood samples were collected from the wing veins of birds in heparinized (100 IU heparin/ml blood) tubes for hematology study.

### Productive performance

Feed and water were supplied *ad libitum* throughout the experiment period. We recorded feed consumption daily throughout the entire experimental period. Feed and water consumption were recorded as grams of feed consumed per day (gm/day/group) and water consumed per day (ml/day/group). Egg production was recorded daily and calculated as hen-day egg production. Eggs were collected and weighed to record the average egg weight every week.

### Determination of Pb concentration in different organs and layers

After collection, samples of bone, gizzard, liver, and heart were placed in a freezer at −4°C until use. All samples were dehydrated, dried at 70°C, and ground at 1,500–2,000 rpm using an electric grinder. Digestion and metal extraction were performed as previously described elsewhere [11]**,** with minor modifications. For the determination of Pb concentration, approximately 0.5 gm of tissue, i.e., bone, gizzard, liver, and heart, was weighed and put into a digestion vessel with 6 ml nitric acid. The samples were then mineralized in a microwave oven into an acid-cleaned digest tube. Then 2.5 ml of 70% nitric acid analytical grade was added to each of the digestion tubes, and all the tubes were labeled, sealed, and left overnight for digestion. After that, 1.5 ml of hydrogen peroxide (30%) of analytical grade was added to each tube. Then the mixture was placed into a digestion system (DS6-1007) and gradually heated in 10°C steps up to a final temperature of 120°C until the vapor and acid fluid inside the vessel turned clear, and this temperature was maintained for 4 h until the digestion was accomplished. The digested solution was then gradually poured into 15 ml falcon tubes and centrifuged at 3,000 rpm for 10 min. The supernatant was separated and made up to 10 ml with deionized water. These solutions were stored at −20°C until analysis. The concentration of Pb was determined using an atomic absorption spectrophotometer with a Pb hollow cathode lamp to determine absorption at 283 nm wavelength in an acetylene-air flame (Shimadzu GFA-7000, Japan).

### Hematological analysis

Erythrocytes were counted by the hemocytometric method on a microscope slide containing a chamber that holds a specified volume of diluted blood. The hemoglobin (Hb) concentration was determined using the hemometer by the acid-hematine method, and the packed cell volume (PCV) was determined by the micro-hematocrit tube method. For erythrocytes, the mean corpuscular Hb concentration (MCHC), the mean corpuscular Hb (MCH), and the mean corpuscular volume (MCV) were calculated using formulas like MCV (µ^3^) = PCV (%)/red blood cell (RBC) (10^6^/mm^3^), MCHC (%) = Hb (gm/dl)/PCV (%), and MCH (pg) = Hb × 10/RBC (10^6^/mm^3^), respectively.

### Statistical analysis

All data were presented as mean ± SEM, and the results were compared statistically by one-way analysis of variance (ANOVA) using Statistical Package for the Social Sciences software. A *p* value of 0.05 or lower is considered statistically significant.

## Results

### Sp reduces the Pb concentration in different tissues

**Figure 1.**
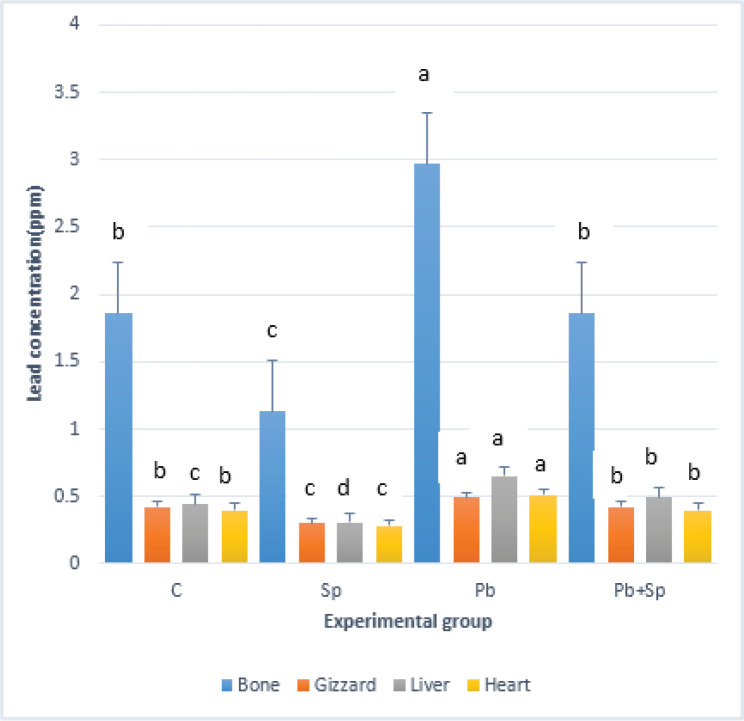
Effect of Sp on reduction of tissue concentration of Pb. Note that Sp treatment significantly reduced Pb concentrations in all organs of the Pb-treated group compared with groups without Sp treatment. Bars with different letters between each group indicated the significant difference at *p* < 0.05 level, data expressed as mean ± SEM (*n =* 5).

Figure 1 demonstrates a significant increase in Pb concentration in various organs, including bone, gizzard, liver, and heart, compared to the control. The Pb concentrations were significantly (*p <* 0.05) higher in the bones, followed by the gizzard, liver, and heart. However, Sp treatment greatly decreased (*p* < 0.05) the amount of Pb in all organs of the Pb-treated group compared to groups that did not receive Sp treatment (Fig. 1).

**Table 1.** The effect of dietary Sp supplement on hematological parameters of birds exposed to Pb for 4 weeks.

Parameters	Group			
	C	Sp	Pb	Pb + Sp
RBC (10^6^/mm^3^)	2.79 ± 0.16^bc^	3.14 ± 0.25^a^	2.58 ± 0.31^c^	2.77 ± 0.15^bc^
Hb (gm/dl)	7.42 ± 0.30^b^	8.98 ± 1.98^a^	6.54 ± 0.62^b^	7.24 ± 0.49^b^
PCV%	25.80 ± 1.92^ab^	29.60 ± 2.60^a^	23.80 ± 3.34^b^	25.80 ± 2.94^ab^
MCV (µ^3^)	92.48 ± 5.9^a^	93.85 ± 9.04^a^	91.96 ± 5.7^a^	92.33 ± 11.2^a^
MCHC (%)	28.94 ± 3.1^a^	29.43 ± 2.5^a^	27.59 ± 2.4^a^	28.21 ± 2.0^a^
MCH (pg)	26.65 ± 1.79^a^	27.83 ± 1.27^a^	25.42 ± 2.2^a^	26.15 ± 1.7^a^

Different letters among groups indicate a significant difference at *p* < 0.05. Data expressed as mean ± SEM (*n* = 10).

**Figure 2.**
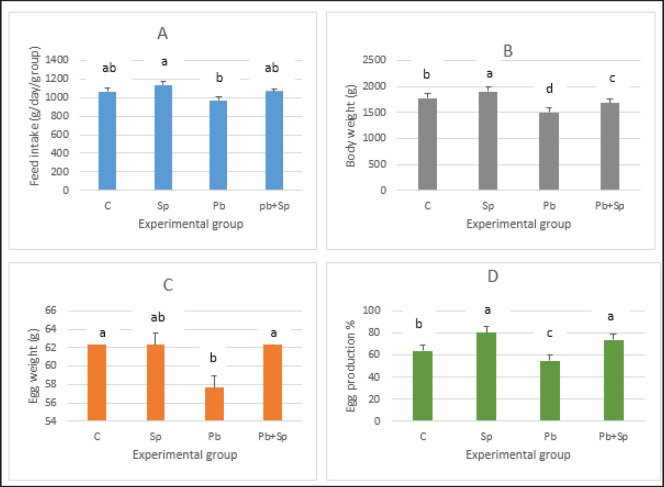
Effect of Sp supplementation on productive performance of Pb-induced laying hens. Feed intake (A), body weight grain (B), egg weight (C), and egg production (D). Bars with different letters within each group indicate a significant difference at *p* < 0.05. Data expressed as mean ± SEM (*n =* 10).

### Effects of Sp on hematological parameters

Hematological parameters observed in the control and experimental groups are shown in Table 1. Hb, PCV, RBC, MCV, and MCHC values were significantly decreased in the Pb-treated group compared to the control group (*p <* 0.05). However, these parameters were significantly increased in the Sp and Pb + Sp groups compared to the Pb group (*p <* 0.05).

Values are represented as the mean ± SEM (*n =* 10). The data were analyzed by a one-way ANOVA. Significantly different at *p <* 0.05. RBCs, Hb concentration, PCV, MCV, MCHC, and MCH. Control (C), Sp, Pb, and Pb + Sp-treated (Pb + Sp).

### Productive performance

The effects of Sp supplementation on the productive performances of Pb-induced laying hens are shown in Figure 2. The overall chicken performance can be categorized as feed consumption, egg production, egg weight, and body weight gain. Although it was not statistically significant, the values of all these parameters tended to increase in the Sp group compared to the basal diet control group. However, the values of the parameters were significantly decreased in the Pb group compared to the control group and the SP group (*p <* 0.05). Interestingly, all parameter values were significantly increased in both the Sp and Pb (Pb + Sp) groups compared to the Pb group only (*p <* 0.05), indicating Sp has a mitigating effect on Pb-induced intoxication in chickens and thus also improves production performance.

## Discussion

Layer hens are used for commercial egg production purposes and are a great source of protein and fat for human consumption. However, high environmental pollutants (such as Pb) negatively affect chicken health and egg production, resulting in severe economic losses in the poultry industry. Therefore, it is crucial to reduce the negative impact of Pb on poultry using sustainable strategies. Sp microalgae is an approved feed supplement for animal consumption in raw, processed, dried, oil, or extract form according to Commission Regulation (EU-No. 575/2011). This microalgae is a sustainable source of energy and is commercially available across the world. It is rich in protein (~65%), carbohydrates (~25%), essential fatty acids (~18%), vitamins, and minerals [12,13]. In addition to the abundance of nutrients, it is also rich in effective bioactive compounds such as phycobiliprotein (β-phycocyanin and c-phycocyanin), β-carotene, phenolic acids, flavonoids, and γ-linoleic acid [10,14]. This microalga also contains pharmacoactive compounds such as alkaloids, glycosides, tannins, steroids, and saponins. These biomolecules are associated with numerous health benefits and act as antioxidants, neoplasia inhibitors, anti-inflammatory mediators, and suppressors of pathogenic bacteria. Moreover, microalgal supplementation in the diet improves the production of key antioxidant enzymes and provides cellular protection [15–17]. As far as we know, no previous studies have examined the effect of Sp supplementation on poultry nutrition under Pb intoxication. Considering the chronic Pb contamination of food, water, and air, Sp may be more suitable for clearing Pb deposits in various tissues and improving egg and meat production.

It has been reported that bioaccumulation of Pb in livestock and poultry can reduce production efficiency, and the highest concentration of Pb accumulates in bone, followed by the kidney and liver, an intermediate concentration in the brain and blood, and the lowest concentration in muscle [18]. Considerable accumulation of Pb in bone has been observed due to the formation of stable Pb-phosphate complexes. Moreover, higher levels of Pb accumulation were observed in laying hens compared to non-laying chickens, and this may be related to higher calcium mobilization in bones during laying. In the present study, we observed that Pb accumulation was significantly increased in various organs compared to controls and higher in bones, followed by the gizzard, liver, and heart. Furthermore, the study showed that body weight and egg production were significantly decreased in the Pb-intoxicated group compared to the control group. These results are in good agreement with previous studies on other heavy metals, including arsenic, cadmium (Cd), and mercury (Hg), which significantly reduce the production performance of livestock and broilers [19]. The mechanism by which Pb reduces egg weight and egg production in adult laying hens is currently unknown and requires further exploration. Bioaccumulation of Pb in visceral organs increases oxidative stress and causes the depletion of antioxidant molecules at the cellular level, which disrupts systemic functions including neurotoxicity, nephrotoxicity, hepatotoxicity, and other mechanisms that result in reduced productive performance [3].

Further, we investigated whether Sp reduces Pb burden in bone, gizzard, liver, and heart; the Pb concentrations in the above-mentioned organs were analyzed. As far as we are aware, the present study showed for the first time that Sp supplementation significantly reduces Pb concentration in all organs in chickens. Although such data are unavailable in chickens, liver Pb accumulation was significantly reduced by dietary SP supplementation in rats [20]. Furthermore, Pb effects on femoral bone were alleviated by Sp supplementation in lactating rats [21]. Similarly, Sp blue-green algae have been found to reduce heavy metals, including cadmium, mercury, arsenic, and nephrotoxic substances, from the body [22].

Pb has been found to cause changes in RBC membranes due to having a high affinity for Pb, which leads to hemolysis or decreased Hb and hematocrit values [23]. Moreover, it has also been reported that hemolytic anemia is frequently observed in cases of chronic Pb intoxication [24]. In the present study, Hb, PCV, RBC, MCV, and MCHC values were significantly decreased in the Pb-intoxicated group compared to the control. However, SP has been reported to improve erythrocyte formation and Hb synthesis and has also been found to adsorb high-degree heavy metals such as Pb, Cd, Zn, and Hg [25]. Moreover, Sp has been shown to reduce the severity of anemia by increasing the concentration of Hb in the blood [26]. Interestingly, in the present study, hematological parameters such as Hb, PCV, RBC, MCV, and MCHC were significantly increased in the Sp and Pb + Sp groups compared to the Pb-only group. Furthermore, body weight, food intake, and egg production were significantly increased in the Sp and Sp + Pb groups compared to the Pb-only group, indicating the protective effect of Sp against Pb. Similar results were observed in rat and carp fish, with Sp improving hematological parameters [27]. Moreover, the beneficial effects of dietary Sp supplementation on the productive performance of poultry have also been reported [28]. On the contrary, accumulation of Pb has been found in the ovary and oviduct with dietary supplementation of Pb, which impairs follicle development and further affects reproductive performance in chickens [5]. It is obvious that Pb exposure induces negative effects on growth, development, and fertility in animals, broilers, and humans [29]. Although the experimental population in the present experiment was small (*n* = 5–10), Sp was shown to reduce Pb-induced negative health and productivity effects, especially in laying hens, a novel finding of our study.

## Conclusion

Dietary supplementation of chickens with Sp to Pb-induced intoxication levels improved their egg production, hematological parameters, and final body weight. These findings suggest that Sp can potentially be used as a natural and effective dietary supplement to mitigate the heavy metal burden in chickens, thereby improving production performance. Further investigation is needed to elucidate the molecular mechanism by which Sp exhibits this protection against heavy metal intoxication.

## List of abbreviations

Pb, Lead; Sp, *Spirulina platensis*; RBC, red blood corpuscle; Hb, hemoglobin; PCV, packed cell volume; MCV, mean corpuscular volume; MCHC, mean corpuscular hemoglobin concentration; MCH, mean corpuscular hemoglobin; pg, picogram; Cd, cadmium; Hg, mercury.
